# Study on Specific Energy Consumption of Rotating Dynamic Filtration for Ship EGC Desulfurization Wastewater Treatment

**DOI:** 10.3390/membranes15120378

**Published:** 2025-12-09

**Authors:** Shiyong Wang, Baohua Yang, Juan Wu, Yanlin Wu, Wenbo Dong

**Affiliations:** 1 Department of Environmental Science and Engineering, Fudan University, Shanghai 200433, China; 2 Shanghai Research Institute of Chemical Industry Co., Ltd., Shanghai 200062, China; wj@ghs.cn; 3 National Petroleum and Chemical Industry Filtration & Separation Engineering Research Center, Shanghai 200062, China; 4 Shanghai Lanke Petrochemical Environmental Protection Technology Co., Ltd., Shanghai 201803, China; 5 Rongsheng Petrochemical Co., Ltd., Hangzhou 311247, China; moqiu_115@163.com; 6 School of Resources and Environmental Engineering, Shanghai Polytechnic University, Shanghai 201209, China

**Keywords:** EGC desulfurization wastewater, rotating dynamic filtration, specific energy consumption, comparison between RDF and CFF, economic analysis

## Abstract

In recent decades, rotating dynamic filtration (RDF) has attracted considerable attention due to its high efficiency and low energy consumption. While most studies have focused on separation behavior and membrane fouling, energy consumption in RDF has received limited attention. This study investigates the specific energy consumption (SEC) of the RDF process for ship exhaust gas cleaning (EGC) desulfurization wastewater treatment and proposes an optimization method based on both energy consumption and equipment cost. The total SEC increases with rotational velocity, circulation flow, feed concentration, and membrane size but decreases with temperature and remains unaffected by the number of membrane elements. In RDF, the total SEC is only 9.05–19.29% of that in tubular cross-flow filtration (CFF) at equivalent shear force ranging from 3.86 Pa to 121.14 Pa. Operating energy and investment costs are primarily determined by the number of membrane elements and the rotational velocity. According to the economic analysis, the lowest treatment cost for EGC wastewater is CNY 6.09 per cubic meter for a 5 m^3^·h^−1^ capacity, using 84 membrane elements (374 mm, 0.2 µm) at a rotational velocity of 200 rpm, an operating pressure of 200 kPa, and a temperature of 40 °C.

## 1. Introduction

Rotating dynamic filtration (RDF) is a novel separation technology that offers excellent performance in fouling control, flux enhancement, and energy savings by utilizing the rotation of the membrane element or other components [1].

Unlike conventional dead-end filtration (DEF) and cross-flow filtration (CFF), RDF achieves relative motion between the membrane surface and feed material through the rotation of the membrane or other components. This motion generates a high shear force, which inhibits concentration polarization and filter cake deposition, with the shear rate typically reaching approximately 3 × 10^5^ s^−1^ [2,3].

In RDF, the movement of the membrane elements or components themselves is used, in contrast to the circulation of large amounts of feed material in CFF. Therefore, the circulation flow in RDF is much lower than that in CFF. Specifically, the RDF feed circulation flow is only 3–5% of the treating capacity in microfiltration (MF) and ultrafiltration (UF), and 10–15% in nanofiltration (NF) and reverse osmosis (RO). In comparison, CFF circulation flow is typically 50–100 times the treating capacity. As a result, RDF can reduce energy consumption by approximately 60–80% compared to CFF [4,5,6].

A new material membrane [7], novel membrane separation methods [8], and other separation techniques based on different mechanisms [9] are the development directions for solving the tough separation problems.

As an alternative to traditional filtration methods, RDF is currently studied and applied in fields such as biology [10,11,12], beverages [13,14,15], and food processing [16,17,18], where filtration is challenging, economic value is high, and processing capacities are small. RDF is also used for the reduction and resource utilization of specific waste liquids [19,20].

Previous research on RDF has mainly focused on permeate flux and retention effects [21,22], filter cake resistance [23], membrane fouling [24,25], optimizing operation [26], and fluid mechanics [27], with limited attention given to energy consumption during the RDF process.

With the growing emphasis on low-carbon and energy-saving policies, RDF offers significant potential due to its low operating energy consumption, despite its relatively high equipment investment. However, at present, there are few systematic studies on the energy consumption and economic performance of RDF, especially regarding the economic analysis and design of its application. Additionally, regarding energy consumption comparison between RDF and CFF, there are only a few qualitative descriptions or simple experimental works rather than theoretical analyses.

This study aims to investigate RDF for the separation of ship exhaust gas cleaning (EGC) desulfurization wastewater, with the goal of clarifying its unique energy consumption characteristics and providing reliable guidance for economic optimization based on both energy consumption and equipment investment. This system study will explore the relationship between specific energy consumption and various influencing factors, including operating parameters (rotational velocity, operating pressure, and feed circulation), feed material properties (concentration, temperature), and the size and number of membrane elements, through laboratory experiments and pilot validation. A theoretical comparison with CFF will be performed based on the equivalent shear force. Eventually, a full life economic analysis, based on operational costs and equipment investment, will be conducted. The related study will support the scientific and economic adoption of RDF.

## 2. Materials and Methods

**Figure 1:**
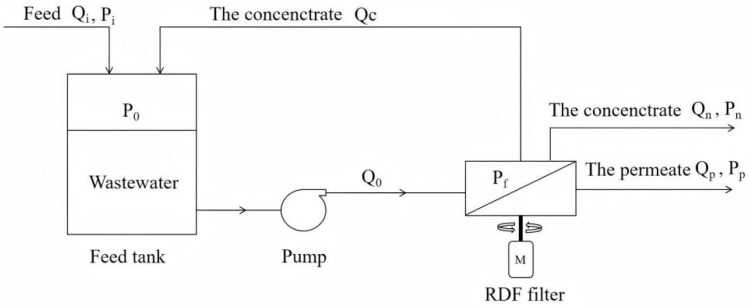
RDF process diagram.

The RDF process, as shown in Figure 1, is similar to CFF, except for the rotation of the membrane element. In addition, the feed circulation flow in RDF is much lower than that in CFF. In CFF, the feed circulation flow is typically 50–100 times the treating capacity, while in RDF, it is only 10%.

**Figure 2:**
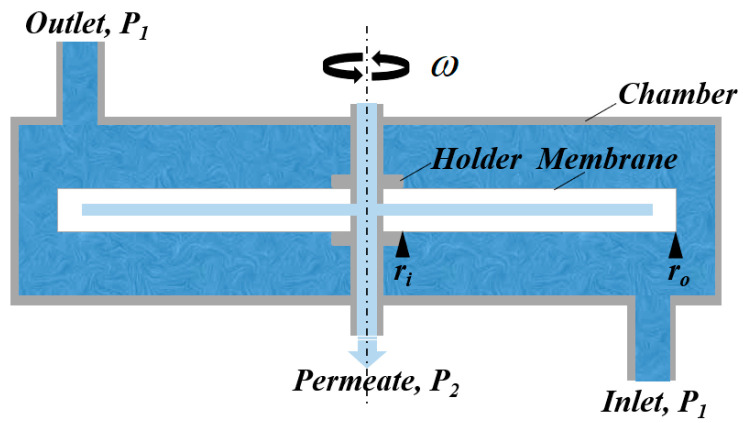
Conceptual diagram of an RDF filter.

**Figure 3:**
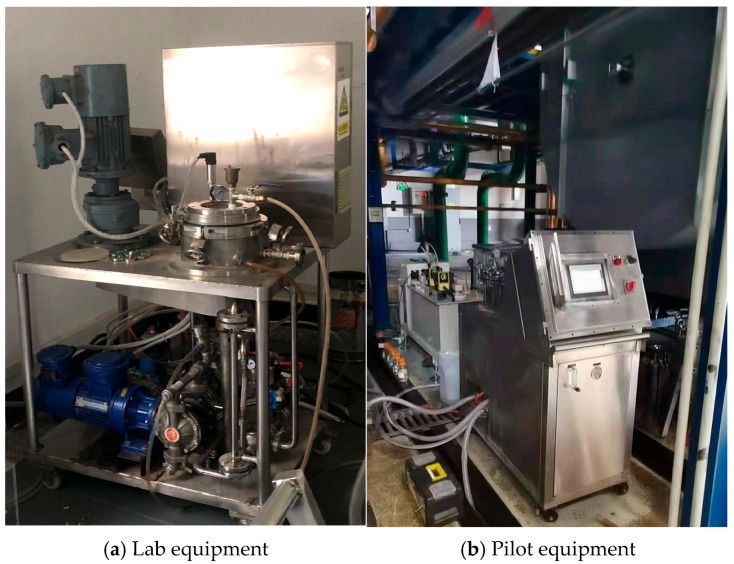
The lab and pilot equipment.

The experimental equipment was composed of a feed tank with stirring and heat exchange facilities, a pump (Zhejiang Alipu Technology Co., Ltd., Jinhua, China), RDF filter, permeate tank, pressure and temperature sensors (Shanghai Hengrui Measurement & Control Technology Co., Ltd., Shanghai, China), a flow meter (Wuxi Jingfan Automation Instrument Co., Ltd., Wuxi, China), valves, pipelines, and a control system. The key part of the system was the RDF filter, comprising a hollow shaft, membrane disc element, and pressure chamber (shown in Figure 2). The ceramic membrane elements from NOVOFLOW (Rain, Germany) were installed on the hollow shaft inside the chamber. The main parameters for each membrane element are listed in Table 1. The membrane rotation was driven by a variable-frequency motor connected to the shaft. As shown in Figure 3, both lab equipment (one 0.035 m^2^ membrane element, 2 L container volume) and pilot equipment (five 0.2 m^2^ membrane elements, 15 L container volume) were utilized. The rotational velocities were from 100 to 900 rpm in the lab tests and from 100 to 600 rpm in the pilot tests.

When the wastewater in the feed tank was pumped into the RDF filter, solid pollutants were retained by the membrane while the permeate passed through. The concentrate was either recirculated to the feed tank or discharged from the filtration system, while the permeate was collected and monitored using the flow meter. To investigate the influence of various factors, such as operating pressure, rotational velocity, temperature, and feed circulation flow, these parameters were controlled by adjusting the pump, motor frequency, and valves.

The total energy consumption in RDF is primarily attributed to the feed circulation pump and the driving motor for membrane element rotation. The pump energy consumption reflects the loss of kinetic energy and static pressure energy of the fluid, while the rotating energy consumption is mostly used to overcome the friction between the membrane surface and the fluid.

The total energy consumption can be expressed as:

(1) $$H_{T}=H_{B}+H_{R}$$

where *H_T_* is the total energy consumption, W; *H_B_* is the pump energy consumption, W; and *H_R_* is the rotating energy consumption, W. The realistic energy consumption of the pump and motor cannot be accurately measured due to the ineffective energy consumption in our experiment. Therefore, the energy consumption of the pump and motor was obtained through theoretical calculation.

The circulating pump energy consumption is calculated by the Bernoulli equation:

(2) $$H_{B}=(P_{f}Q_{0}-P_{P}Q_{P}-P_{n}Q_{n}-P_{0}Q_{C})+h_{f}$$

where *P*_f_ is the operating pressure, kPa; *P*_P_ is the pressure of the permeate side, kPa; *P*_n_ is the pressure of the discharged concentrate, kPa; *P*_0_ is the pressure of the recirculated concentrate, kPa; *Q*_0_ is the pump circulation flow rate, m^3^·h^−1^; *Q*_P_ is the flow rate of the permeate, m^3^·h^−1^; *Q*_n_ is the flow rate of the discharged concentrate, m^3^·h^−1^; *Q*_C_ is the flow rate of the recirculated concentrate, m^3^·h^−1^; and *h*_f_ is the energy loss of pipes and fittings, W. If static head neglected, the calculated value should be slightly larger.

Both the permeate and the concentrate were collected into an open container. Accordingly, *P*_n_, *P*_P_, and *P*_0_ were all atmospheric pressures. Furthermore, *h*_f_ was too small to be ignored, and Equation (2) can be simplified as follows:

(3) $$H_{B}=P_{f}Q_{0}$$

On the other hand, the rotating energy consumption in RDF can be calculated by the following equation [28].

(4) $$H_{R}=2\pi n\int_{0}^{R}\left(\tau_{u}+\tau_{d}\right)r^{2}\omega dr$$

where *n* is the number of membrane elements; *τ_u_* and *τ_d_* are the shear forces on two surfaces of a membrane element, Pa; *τ_u_* and *τ_d_* are the same in this experiment; *r* is the radius of the membrane disc, m; and *ω* is the rotational velocity of the membrane element, rad·s^−1^.

The shear force in the RDF system can be estimated under laminar or turbulent conditions [29] using the following expressions:

(5) $$\tau_{l}=1.81\rho {\left(k\omega \right)}^{3/2}r\nu^{1/2}$$

(6) $$\tau_{t}=0.057\rho {\left(k\omega \right)}^{9/5}r^{8/5}\nu^{1/5}$$

where *τ_l_* is the shear force in laminar flow, Pa; *τ_t_* is the shear force in turbulent flow, Pa; *k* is the velocity factor; *ρ* is the liquid density, kg·m^−3^; *ω* is the rotating angular velocity of the membrane element, rad·s^−1^; *r* is the radius of the membrane element, m; and *ν* is kinematic viscosity, m^2^·s^−1^. When *Re* is less than 2.50 × 10^5^, the flow state is laminar, while when *Re* is greater than 2.50 × 10^5^, the flow state is turbulent.

The shear force on the smooth channel wall of the tubular membrane in the CCF system can be estimated [30].

(7) $$\tau =0.5f\rho u^{2}$$

where *τ* is the shear force on membrane channel wall, Pa; *f* is the friction coefficient, when *Re* ≤ 2400, *f* is equal to 16*Re*^−1^, while 10^5^ ≤ *Re* ≤ 2400, *f* is equal to 0.079*Re*^−0.25^; *ρ* is the wastewater density, g·cm^−1^; and *u* is the linear velocity in the membrane channel surface, m·s^−1^.

The wastewater viscosity is 1.33 mPa·s (20 °C) and 0.86 mPa·s (40 °C), and the density remains almost constant at 0.98 g·cm^−3^ (20 °C and 40 °C).

Specific energy consumption (SEC), defined as the energy consumed per unit treating volume, is used to evaluate the process’s energy consumption. It is given by:

(8) $$U_{R}=\frac{H}{V}$$

where *U_R_* is the SEC, W·m^−3^; *H* represents the energy consumption, W; and *V* represents the permeate volume obtained, m^3^. The permeate volume can be easily calculated from the flux and membrane area in the experiment. A reduced SEC correlates with lower energy costs, which can significantly improve profitability.

## 3. Results and Discussion

### 3.1. Experimental Study on SEC in the RDF Process

#### 3.1.1. Influence of Operating Parameters

Energy consumption is a key factor in the RDF process and is influenced by various operating parameters, such as rotational velocity, operating pressure, and feed circulation flow.

In this study, SEC was investigated at rotational velocities ranging from 100 to 900 rpm, using one 1# membrane disc under conditions with a feed concentration of 266 mg·L^−1^, a circulation flow rate of 60 L·h^−1^, pressures of 100 kPa and 300 kPa, and a temperature of 20 °C. The experimental fluxes with rotating velocity at 100 kPa and 300 kPa are presented in Table 2.

The pump energy consumption was calculated using Equation (3), where *P*_f_ is either 100 kPa or 300 kPa and *Q*_0_ is 60 L·h^−1^. The rotating energy consumption was calculated by evaluating the shear force, an important parameter determined using Equations (5) and (6) from our previous study. As for the value of the velocity factor, it reflects the deviation between theory and practice. The back pressure values under different rotational velocities were measured through the designed experiment and then compared with the theoretical back pressure values calculated. Eventually, *k* was calculated based on the difference between these two values [31].

Furthermore, the total SEC, pump SEC, and rotating SEC can be estimated with Equation (8) based on the experimental flux data. The subsequent calculations of energy consumption and SEC are similar to the above method.

**Figure 4:**
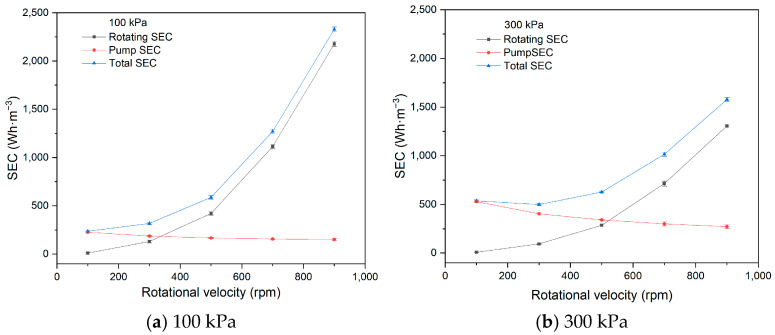
SEC with rotating velocity (100 kPa, 300 kPa).

As depicted in Figure 4, the pump SEC decreases with increasing rotational velocity, while both the rotating SEC and total SEC increase at both 100 kPa and 300 kPa.

At constant feed circulation flow, the pump energy consumption remains unchanged, but the permeate flux increases with higher rotational velocities, leading to a reduction in the pump SEC at both 100 kPa and 300 kPa.

The rotation of the membrane element is a unique factor in RDF, directly influencing both the separation efficiency and energy consumption. Increasing the rotational velocity enhances the shear force at the membrane surface, alleviating concentration polarization and filter cake, reducing filtration resistance, and increasing flux. However, as rotational velocity increases, rotating energy consumption, calculated using Equation (4), also rises. In this study, the increase in rotating energy consumption outweighs the increase in permeate flux, leading to a rise in the rotating SEC. Additionally, since the rotating SEC is greater than the pump SEC, the total SEC increases with higher rotational velocities.

Experiments were also conducted at operating pressures ranging from 100 kPa to 300 kPa, using the 1# membrane, a feed circulation flow rate of 60 L·h^−1^, a concentration of 266 mg·L^−1^, a temperature of 20 °C, and rotational velocities of 100 and 700 rpm. The data of the permeate flux are presented in Table 3.

**Figure 5:**
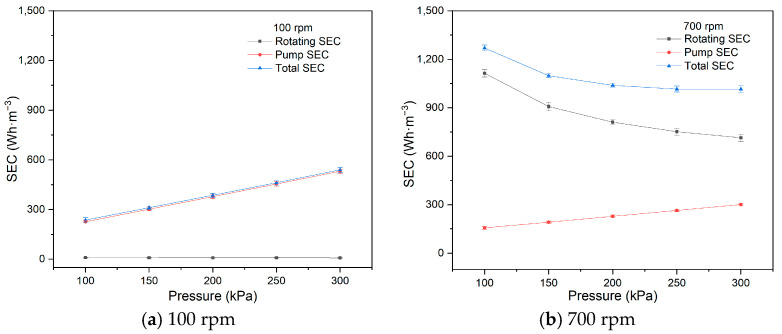
SEC with pressure (100 rpm, 700 rpm).

Figure 5 shows the relationship between SEC and operating pressure at 100 rpm and 700 rpm. At 100 rpm, the pump SEC is higher than the rotating SEC and increases with increasing operating pressure, while the rotating SEC decreases slightly. Therefore, the total SEC follows a similar trend to that of the pump SEC at 100 rpm. However, at 700 rpm, the rotating SEC is higher than the pump SEC and decreases as operating pressure increases. The total SEC is primarily determined by the rotating SEC and decreases with increasing operating pressure.

Operating pressure serves as the driving force in the RDF process, with both flux and permeate volume increasing as pressure rises. The energy consumed by the pump is directly proportional to operating pressure and feed circulation. In this study, the increase in pump energy consumption was greater than the increase in flux, resulting in an increase in SEC with higher operating pressure.

The energy consumed by the driving motor for membrane rotation remains constant at the same rotational velocity. Therefore, increasing operating pressure leads to a higher permeate volume and a reduction in the rotating SEC. Moreover, at 100 rpm, the pump SEC dominates, while at 700 rpm, the rotating SEC becomes the dominant factor. As rotational velocity increases, the energy consumption of membrane rotation exceeds that of the pump. In practical RDF applications, where more membrane elements and higher rotational velocities are typically required, the rotating SEC becomes the governing factor. Additionally, increasing operating pressure benefits energy conservation at higher rotational velocities.

The influence of different feed circulation flow rates, ranging from 20 L·h^−1^ to 100 L·h^−1^, on SEC was also investigated at a concentration of 266 mg·L^−1^, a temperature of 20 °C, a pressure of 200 kPa, and rotational velocities of 100 rpm and 700 rpm, using the 1# membrane element. The fluxes with different circulation flows are shown in Table 4 below.

**Figure 6:**
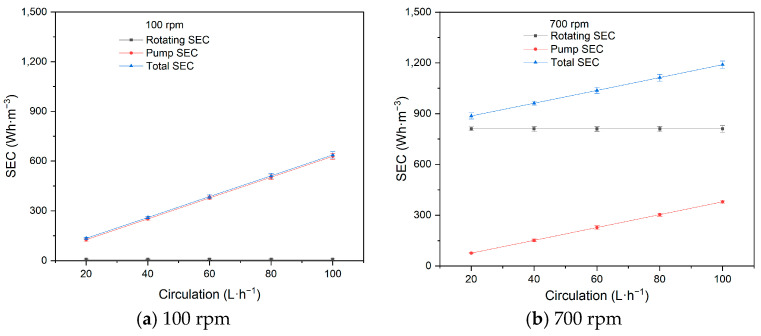
Influence of circulation flow on SEC (100 rpm, 700 rpm).

As shown in Figure 6, the SEC with varying circulation flow exhibits similar patterns at both 100 rpm and 700 rpm.

As circulation flow increases, both the pump SEC and the total SEC increase, while the rotating SEC remains relatively unchanged at both 100 rpm and 700 rpm. The energy consumed by the driving motor for membrane rotation does not change under constant rotational velocity. However, the energy consumption of the pump is directly proportional to the circulation flow. Based on our previous experiments, the circulation flow has a negligible impact on flux, so the rotating SEC remains nearly constant. On the other hand, the pump SEC increases due to the rise in the circulation flow and associated energy consumption. The purpose of the circulation flow is to ensure process circulation and homogenization of feed material within the RDF system. Since the circulation flow is typically less than 10% of the treating capacity, its impact on overall energy consumption is minimal, particularly at high rotational velocities.

#### 3.1.2. Influence of Material Properties

The characteristics of the feed material, such as feed concentration and temperature, also influence energy consumption in the RDF process.

The effect of feed concentration on SEC was investigated at a feed circulation flow rate of 60 L·h^−1^, a temperature of 20 °C, an operating pressure of 200 kPa, and rotational velocities of 300 rpm and 700 rpm, using the 1# membrane element. The fluxes with different concentrations are shown in Table 5.

**Figure 7:**
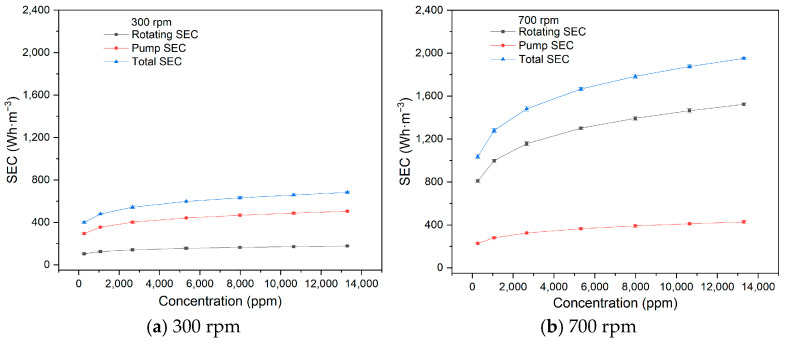
Influence of concentration on SEC (300 rpm, 700 rpm).

As shown in Figure 7, the pump SEC, rotating SEC, and total SEC all increase steadily with feed concentration at both 100 rpm and 700 rpm. At 100 rpm, the pump SEC accounts for a larger proportion of the total, while the rotating SEC dominates at 700 rpm. When operating pressure, circulation flow, rotational velocity, temperature, and membrane size are fixed, both pump energy consumption and rotating energy consumption remain stable, with SEC dependent solely on permeate flux. As feed concentration increases, more solid particles are intercepted on the membrane surface, resulting in a reduction in permeate flux and an increase in all SEC values. However, the particle layer deposited on the membrane surface does not accumulate indefinitely. The deposition and reverse diffusion of particles back into the feed stream will reach an equilibrium at a critical concentration. Therefore, the reduction in permeate flux and the increase in SEC become less significant at higher concentrations.

Temperature fluctuations are common in practical applications. To examine the effect of temperature on SEC, experiments were conducted from 20 °C to 60 °C using a feed concentration of 266 ppm, a feed circulation flow rate of 60 L·h^−1^, an operating pressure of 200 kPa, and rotational velocities of 100 rpm and 700 rpm, with the 1# membrane element. The fluxes with temperature are shown in Table 6.

**Figure 8:**
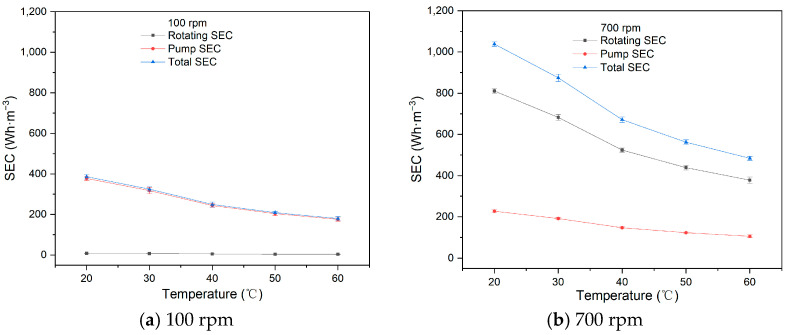
Influence of temperature on SEC.

As shown in Figure 8, the pump SEC, rotating SEC, and total SEC all decrease with increasing temperature at both 100 rpm and 700 rpm. The total SEC decreases from 386.37 Wh·m^−3^ at 20 °C to 180.11 Wh·m^−3^ at 60 °C at 100 rpm, and from 1038.37 Wh·m^−3^ at 20 °C to 484.05 Wh·m^−3^ at 60 °C at 700 rpm.

When operating pressure, circulation flow, membrane size, and rotational velocity are held constant, both pump and rotating energy consumption remain fixed. Therefore, SEC is directly related to permeate flux. As temperature increases, the viscosity of the wastewater decreases, accelerating particle reverse diffusion, reducing separation resistance, and improving permeate flux. Therefore, SEC gradually decreases with increasing temperature. If feasible, operating the RDF process at higher temperatures for EGC wastewater filtration can lower operational costs. But, according to the IMO MEPC.259(68) requirement [32], the discharged desulfurization wastewater must not exceed 60 °C to avoid affecting marine life. The temperature of the desulfurization wastewater remains around 40–50 °C in the actual process; no further consideration is given to the heating of wastewater.

#### 3.1.3. Influence of Membrane Element

The membrane element is a critical component, and its size and quantity can affect energy consumption in the RDF process. We studied two membrane sizes: 152 mm diameter (1# membrane, at 60 L·h^−1^ feed circulation) and 374 mm diameter (2# membrane, at 350 L·h^−1^ feed circulation). These experiments were conducted under conditions of 266 ppm feed concentration, 20 °C temperature, 100 kPa and 300 kPa operating pressures, and rotational velocities from 100 rpm to 900 rpm. The fluxes with rotational velocity for the 347 mm and 152 mm membranes are shown in Table 7 and Table 8.

Regarding the threshold value of the Reynolds number in hydrodynamics, the 374 mm membrane requires 100 rpm to reach 2.50 × 10^5^ in turbulent flow, while the 152 mm membrane reaches the value at 560 rpm. There is a gradient distribution of shear force in the radial direction of membrane elements in RDF, in which the smallest value appears at the inner section and the largest occurs at the disc edge. The larger the membrane size, the greater the shear force on the membrane surface. The maximum value of the 152 mm membrane is 27.05 Pa, while the maximum value of the 374 mm membrane is 128.64 Pa at 300 rpm.

**Figure 9:**
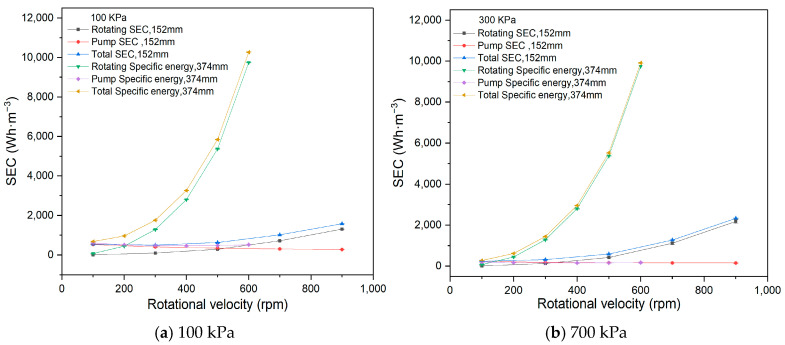
Influence of membrane size on SEC.

As shown in Figure 9, the rotating SEC and total SEC for the 374 mm membrane are significantly higher than those for the 152 mm membrane at the same rotational velocity. At a pressure of 100 kPa and a rotational velocity of 300 rpm, the total SEC for the 374 mm membrane is 1440.66 Wh·m^−3^, while for the 152 mm membrane, it is 316.79 Wh·m^−3^. Larger membrane sizes result in greater friction, leading to higher energy consumption due to increased frictional resistance. However, a larger membrane size also increases permeate flux due to higher shear forces on the membrane surface. In this study, the increase in energy consumption was more pronounced than the increase in permeate flux, causing the rotating SEC and total SEC to rise with membrane size. In addition, compared to the rotating SEC, the pump SEC for the 374 mm membrane was much smaller and could often be considered negligible.

In actual RDF equipment, multiple membrane discs are mounted on a hollow shaft. In the previous laboratory experiments, a single membrane element was used due to the small scale of the system. To further understand the relationship between membrane number and energy consumption, experiments were conducted under conditions of 266 ppm feed concentration, 20 °C temperature, 60 L·h^−1^ feed circulation, and 250 kPa pressure. The rotational velocities from 100 rpm to 900 rpm lead to a flux from 0.3403 m^3^·m^−2^·h^−1^ to 0.4940 m^3^·m^−2^·h^−1^.

**Figure 10:**
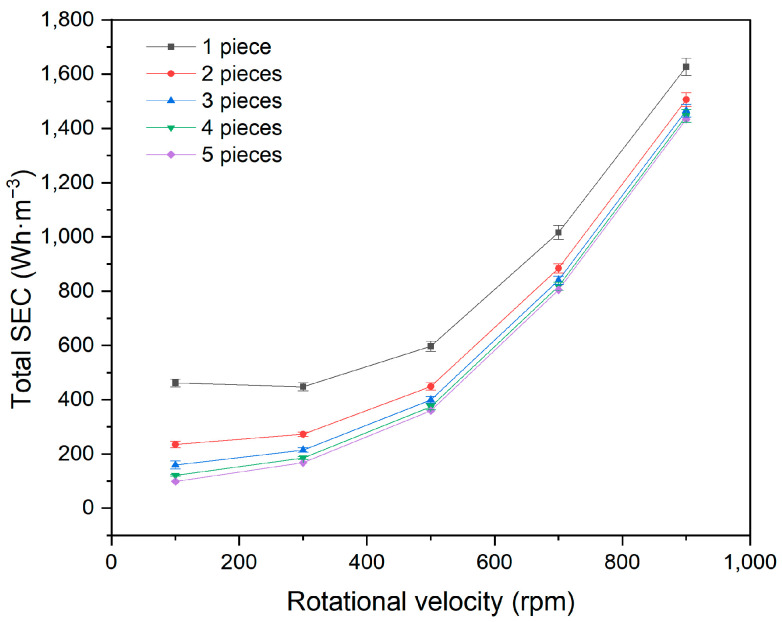
Influence of membrane element numbers on total SEC.

Figure 10 shows that the total SEC decreases with the number of membrane discs. However, the rate of decrease slows as the number of membrane elements increases, with the total SEC for four membranes nearly equal to that of five membranes.

The total energy consumption in the RDF process is the sum of the pump energy consumption and the rotating energy consumption. The pump energy consumption is related only to circulation flow and operating pressure. When these factors remain constant, the pump energy consumption does not change. However, the energy consumption for driving membrane rotation increases proportionally with the number of membrane elements, as described by Equation (4), resulting in higher total energy consumption.

With a larger number of membrane elements, the energy consumption of the pump becomes negligible, and the total energy consumption is primarily determined by the energy used for membrane rotation. Although the total energy consumption increases, the permeate volume also increases proportionally. Therefore, SEC remains approximately constant with the number of membrane elements. In practical applications, the energy consumption per unit of treated volume is not significantly affected by the number of membrane elements.

### 3.2. Comparison of Energy Consumption Between RDF and CFF

In order to facilitate the comparison of energy consumption between RDF and CFF, some simplified assumptions were made in this study. Usually, the flux is a result of shear force and membrane fouling in the separation process. It is assumed that the equivalent shear force leads to an identical flux based on similar membrane fouling in RDF and CFF.

The area of the 1# membrane disc in RDF is 0.035 m^2^ for EGC wastewater treatment in lab tests. To match an equal membrane area, the 19-channel tubular ceramic membrane selected for CFF had a length of 178 mm and a channel diameter of 3.3 mm. Energy consumption comparisons between RDF and CFF were carried out under the following conditions: a wastewater concentration of 266 ppm, a temperature of 20 °C, a feed circulation of 60 L·h^−1^, an operating pressure of 300 kPa, and rotational velocities of 100 rpm, 300 rpm, 500 rpm, 700 rpm, and 900 rpm. The flux refers to the data at 300 kPa in Table 2.

The pump SEC, the rotating SEC, and the total SEC in RDF were already determined in the former part of this study. In addition, the equivalent shear forces with rotational velocities in RDF were also obtained.

Based on the same equivalent shear forces, the linear flow velocity in CFF was derived from Equation (7); then, the circulation flow was calculated by linear velocity and the total cross-sectional area of channels. As shown in Table 9, the circulation flow in CFF is much higher than the feed circulation flow of 60 L·h^−1^ in RDF.

Furthermore, the CFF energy consumption was determined using Equation (3), where the operating pressure, circulation flow are known. Finally, the total SEC in CFF was obtained by Equation (8), in which both the energy consumption and the flux are the known parameters.

As presented in Table 10, the SEC for RDF is significantly lower than that of CFF, typically around 10–20% of the CFF value. RDF achieves shear on the membrane surface through the rotation of the membrane disc itself, while CFF generates shear by the high-velocity flow created by feed circulation. Most of the energy was consumed by rotation driving in RDF and by liquid circulating in CFF. The large circulation flow in CFF is the primary reason for its higher energy consumption. In turbulent flow conditions, energy consumption is proportional to the cube of the fluid velocity.

As circulation flow increases, energy consumption rises. To maintain effective shear on the membrane surface, a large-capacity circulation pump is required, which brings the risk of equipment failure.

A new method for comparing energy consumption based on equivalent shear force was proposed, which is a helpful reference for actual energy consumption analysis. However, regarding fouling, the shape of the membrane, the distribution of shear force, and the filter cake, further research is still needed in the future.

### 3.3. Study on SEC in the Pilot Experiment

A continuous-mode pilot experiment was conducted at a feed concentration of 183 mg·L^−1^, a feed circulation rate of 350 L·h^−1^, a rotational velocity of 350 rpm, an operating pressure of 250 kPa, and a temperature of 40 °C, using the 2# membrane element.

The industrial requirements are concentrated 20 times (yield of 95.0%) for EGC wastewater separation. In this study, the concentration was increased by 30 times (yield of 96.6%), or even higher, and the permeate flux did not reduce significantly, indicating that RDF exhibited good anti-pollution ability. On the other hand, the yield increase was limited, only 1.6% from the concentration ratio of 20 to 30, so the concentration multiple of wastewater was kept at 30-fold in the pilot test.

Due to the limitations of the experimental conditions, three pilot tests totaling 45 h were conducted. After each experiment, the membrane was cleaned using a feasible cleaning method and then reused. The fouling membrane was soaked in 2% (wt) sodium hydroxide solution for 2 h, 4000 ppm sodium dodecyl sulfonate for 2.5 h, and then 2% (wt) nitric acid for 2.5 h at 40 °C. The flux decreased from 0.3305 m^3^·m^−2^·h^−1^ to 0.3078 m^3^·m^−2^·h^−1^ during the first test (24 h), the flux declined from 0.3308 m^3^·m^−2^·h^−1^ to 0.3196 m^3^·m^−2^·h^−1^ in the second test (12 h), and the flux went down form 0.3287 m^3^·m^−2^·h^−1^ to 0.3254 m^3^·m^−2^·h^−1^ in the third test (9 h). It was observed that the flux was relatively stable in the pilot tests, and the membrane fouling was not severe. Nevertheless, a further verification study should be conducted in long-term industrial operation.

**Figure 11:**
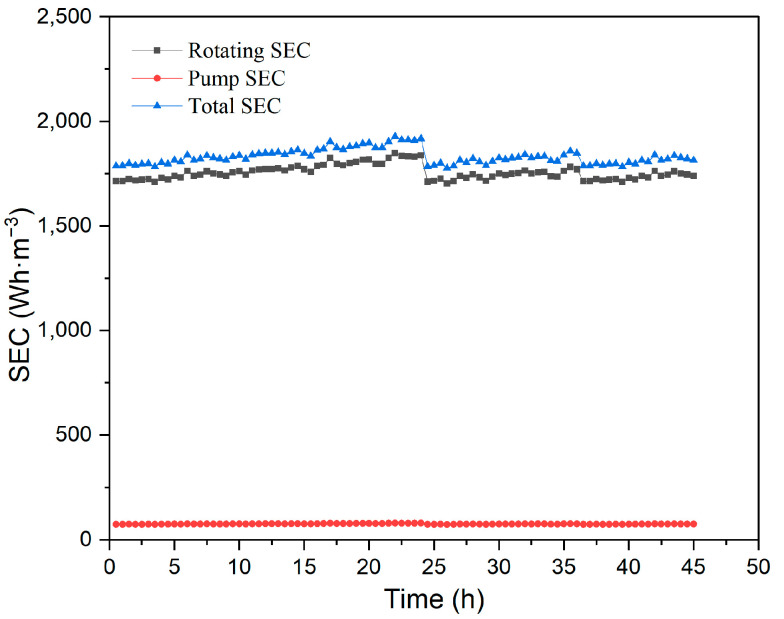
SEC in the continuous pilot test process.

As shown in Figure 11, the pump SEC, rotating SEC, and total SEC remained stable throughout the pilot experiment, with the rotating SEC accounting for a large proportion of the total SEC. The average total SEC was 1830.86 Wh·m^−3^. If membrane fouling becomes more severe in actual application, the flux will fall, while the SEC will increase.

### 3.4. Optimized Design of the RDF System

In addition to meeting the separation requirements, cost is a critical consideration, including both equipment and operating costs.

The equipment cost is primarily influenced by the scale of the system, the number of membrane elements, and the degree of automation, among other factors. Operating costs are directly affected by parameters such as rotational velocity, operating pressure, operating temperature, feed concentration, and concentration ratio. In practical applications, the temperature, concentration, and concentration ratio are generally fixed, making operating pressure and rotational velocity the most significant factors influencing cost.

Operating pressure mainly impacts the operating cost, while rotational velocity affects both operating and equipment costs. Therefore, this study considers both equipment investment and operating costs at different rotational velocities.

Based on the pilot treatment of desulfurization wastewater, an economic analysis was conducted for rotational velocities ranging from 100 rpm to 600 rpm under the following conditions: 5 m^3^·h^−1^ capacity, 3660 ppm feed concentration (30-fold concentration), a 30-times concentrated ratio, 200 kPa operating pressure, and a temperature of 40 °C.

As shown in Table 11, for a 5 m^3^·h^−1^ capacity, increasing rotational velocity led to higher energy consumption, but a decrease in the number of membrane elements and filtration area was required to maintain improved flux. As the required number of membranes decreased from 104 to 67, the rotational velocity was increased from 100 rpm to 600 rpm, and the energy consumption rose from 0.69 kWh to 31.09 kWh.

The reference electricity price of CNY 2 per kWh was based on shipboard operating scenarios; the membrane cost of CNY 50,000 per square meter of membrane was estimated based on engineering experience. A 4-year service life and an annual operation time of 8000 h are the general requirements of customers. Moreover, there is almost no scaling effect for the sake of the same-sized membrane used.

**Figure 12:**
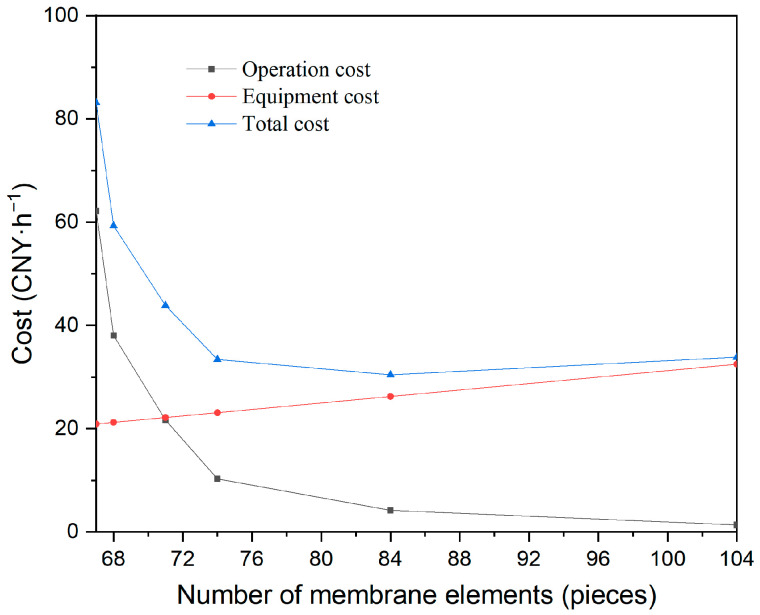
Influence of the number of membrane elements on wastewater treatment cost.

As depicted in Figure 12, as the number of membrane elements increased, the required rotational velocity decreased, resulting in higher operating costs but lower equipment costs. When 104 membrane elements were used, the operating cost was only 1.37 CNY·h^−1^, but the corresponding equipment investment was 32.5 CNY·h^−1^. An optimal point exists where the total cost (the sum of operating and investment costs) is minimized. For a 5 m^3^·h^−1^ EGC wastewater treatment system, 84 membrane elements and a rotational velocity of 200 rpm provide the lowest total cost, at 30.44 CNY·h^−1^, or CNY 6.09 per cubic meter of wastewater.

Meanwhile, the basic cost data for economic analysis should be adjusted according to the actual application. When the membrane cost increases, the rotational velocity will elevate, and the number of membranes will decrease. The rotational velocity will fall, and the number of membranes will increase with longer membrane lifetimes and higher electricity prices on the ship.

The optimization method can be applied to larger capacities because of an insignificant scaling effect with the same-sized membrane element used. Moreover, the larger capacities can also be met through the parallel design. As for ship desulfurization wastewater treatment, one filter with 84 pieces of membrane discs was for 5 m^3^·h^−1^, while four filters with 84 pieces were for 20 m^3^·h^−1^.

## 4. Conclusions

This study aimed to enhance our understanding of energy consumption in the RDF process and provide an efficient, feasible cost-optimization method for EGC wastewater treatment, even for the separation of dairy products, microalgae, emulsions, etc.

SEC, defined as the energy consumed per permeate volume, was used to evaluate the energy consumption of the process. The total SEC is the sum of the pump SEC and the rotating SEC, with the pump SEC typically accounting for a small proportion of the total; in some cases, it can even be neglected. The total SEC increased with higher rotational velocity, circulation flow, feed concentration, and membrane size, while it decreased with rising temperature. The total SEC was unaffected by the number of membrane elements. Additionally, in the pilot experiment, the total SEC, pump SEC, and rotating SEC remained stable, with an average total SEC of 1830.86 Wh·m^−3^, where the pump SEC was negligible.

The theoretical comparison with CFF was first performed based on the equivalent shear force. RDF is an energy-efficient technology, with its SEC ranging from 9.05 to 19.29% of that of tubular CFF at equivalent shear force ranging from 3.86 Pa to 121.14 Pa.

The operational and investment costs are primarily influenced by the number of membrane elements and the rotational velocity. Based on the optimization design, the lowest treatment cost was CNY 6.09 per cubic meter for a 5 m^3^·h^−1^ capacity, using 84 membrane elements (374 mm, 0.2 µm) at a rotational velocity of 200 rpm, an operating pressure of 200 kPa, and a temperature of 40 °C.

Overall, this study provides a deeper understanding of RDF energy consumption, offers a novel optimization approach based on both energy consumption and investment costs, and supports the scientific and economic applications of RDF.

## Figures and Tables

**Table 1:** Main parameters of membrane elements in this study.

Sample No.	Precision (μm)	Diameter (mm)	Area (m^2^)	Resistance (10^10^ m^−1^)
1#	0.2	152	0.035	42.94
2#	0.2	374	0.20	41.93

**Table 2:** Flux with different rotating velocities (100 kPa, 300 kPa).

Rotating Velocity (rpm)	Flux at 100 kPa (m^3^·m^−2^·h^−1^)	Flux at 300 kPa (m^3^·m^−2^·h^−1^)
100	0.2096	0.2694
300	0.2558	0.3532
500	0.2849	0.4180
700	0.3041	0.4742
900	0.3144	0.5242

**Table 3:** Flux with operating pressure (100 rpm, 700 rpm).

Operating Pressure (kPa)	Flux at 100 rpm (m^3^·m^−2^·h^−1^)	Flux at 700 rpm (m^3^·m^−2^·h^−1^)
100	0.2102	0.3042
150	0.2360	0.3733
200	0.2518	0.4178
250	0.2621	0.4506
300	0.2684	0.4743

**Table 4:** Flux with circulation flow (100 rpm, 700 rpm).

Circulation Flow (L·h^−1^)	Flux at 100 rpm (m^3^·m^−2^·h^−1^)	Flux at 700 rpm (m^3^·m^−2^·h^−1^)
20	0.2518	0.4178
40	0.2524	0.4181
60	0.2519	0.4182
80	0.2523	0.4182
100	0.2521	0.4179

**Table 5:** Flux with concentration (300 rpm, 700 rpm).

Concentration (ppm)	Flux at 300 rpm (m^3^·m^−2^·h^−1^)	Flux at 700 rpm (m^3^·m^−2^·h^−1^)
266	0.3222	0.4183
1064	0.2683	0.3391
2660	0.2365	0.2928
5320	0.2151	0.2603
7980	0.2037	0.2431
10,640	0.1952	0.2313
13,300	0.1883	0.2223

**Table 6:** Flux with temperature (100 rpm, 700 rpm).

Temperature (°C)	Flux at 100 rpm (m^3^·m^−2^·h^−1^)	Flux at 700 rpm (m^3^·m^−2^·h^−1^)
20	0.2518	0.4178
30	0.2990	0.4961
40	0.3894	0.6461
50	0.4651	0.7718
60	0.5402	0.8962

**Table 7:** Flux with rotational velocity of 152 mm membrane (100 rpm, 300 rpm).

Rotational Velocity (rpm)	Flux at 100 rpm (m^3^·m^−2^·h^−1^)	Flux at 300 rpm (m^3^·m^−2^·h^−1^)
100	0.2096	0.2694
300	0.2558	0.3532
500	0.2849	0.4180
700	0.3041	0.4742
900	0.3144	0.5242

**Table 8:** Flux with temperature of 374 mm membrane (100 rpm, 300 rpm).

Rotational Velocity (rpm)	Flux at 100 rpm (m^3^·m^−2^·h^−1^)	Flux at 300 rpm (m^3^·m^−2^·h^−1^)
100	0.2420	0.3242
200	0.2846	0.4136
300	0.3074	0.4833
400	0.3147	0.5406
500	0.3065	0.5878
600	0.2814	0.6254

**Table 9:** Equivalent shear force and circulation flow in CFF.

No.	Rotational Velocity in RDF (rpm)	Equivalent Shear Force (Pa)	Linear Velocity in CFF (m·s^−1^)	Circulation Flow in CFF (L·h^−1^)
1	100	3.86	0.81	339.14
2	300	19.13	2.02	1017.43
3	500	41.16	3.13	1701.56
4	700	77.70	4.50	2379.84
5	900	121.14	5.80	3058.13

**Table 10:** Energy consumption comparison between RDF and CFF.

No.	Equivalent Shear Force (Pa)	Total SEC of CFF (Wh·m^−3^)	Total SEC of RDF (Wh·m^−3^)	Total SEC Ratio of RDF to CFF (%)
1	3.86	2787.21	537.74	19.29
2	19.13	5301.56	498.79	9.41
3	41.16	6940.79	628.40	9.05
4	77.70	8796.57	1015.19	11.54
5	121.14	10,255.73	1578.04	15.39

**Table 11:** Energy consumption with rotational velocities and membrane numbers.

No.	Rotational Velocity (rpm)	Total SEC In RDF (Wh·m^−3^)	Energy Consumption (kWh)	Filtration Area (m^2^)	Number of Elements (PCS)
1	100	144.56	0.69	21	104
2	200	441.24	2.10	17	84
3	300	1083.26	5.15	15	74
4	400	2284.53	10.85	14	71
5	500	4006.20	19.03	13	68
6	600	6546.13	31.09	13	67

## Data Availability

All relevant data supporting the findings of this study are openly available in the Journal of Membranes.
